# Correction to “Clinical, Endocrine and Neuroimaging Findings in Girls With Central Precocious Puberty”

**DOI:** 10.1210/clinem/dgac710

**Published:** 2022-12-13

In the above-named article by Fava D, Calandrino A, Calevo MG, Allegri AEM, Napoli F, Gastaldi R, Patti G, Casalini E, Bassi M, Accogli A, Alyasin ARAA, Ramaglia A, Rossi A, Maghnie M, Morana G, and Di Iorgi N (*J Clin Endo Metab*. 2022; 107(10): e4132–e4143; doi: 10.1210/clinem/dgac422), a funder was left out of the Acknowledgments:

The following sentence should appear in the Acknowledgments: “This work was supported by the Italian Ministry of Health – Ricerca corrente 2022.”

The article has been corrected online.

doi: 10.1210/clinem/dgac422

